# The silence of lost smiles: multilevel analysis of edentulism among older adults in contextual and individual interfaces in Brazil (SB Brasil 2023)

**DOI:** 10.1590/1980-549720260028.supl.1

**Published:** 2026-07-27

**Authors:** Rafael da Silveira Moreira, Camila Caroline da Silva, Lucas Fernando Rodrigues dos Santos, Tainá Faustino Mafra, Herika de Arruda Mauricio

**Affiliations:** I Fundação Oswaldo Cruz, Instituto Aggeu Magalhães, Department of Public Health – Recife (PE), Brazil.; II Universidade Federal de Pernambuco, School of Medicine – Recife (PE), Brazil.; III Universidade de Pernambuco, School of Dentistry – Recife (PE), Brazil.

**Keywords:** Tooth loss, Aged, Public health dentistry, Oral health, Dental health surveys

## Abstract

**Objective:**

To analyze contextual and individual factors associated with edentulism among older adults aged 65–74 years in Brazil, based on data from the SB Brasil 2023 national survey.

**Methods:**

A cross-sectional study was conducted with 9,745 participants from the 27 Federative Units. Data collection was conducted using standardized questionnaires and clinical examinations. Contextual municipal variables and individual demographic, socioeconomic, service utilization variables were analyzed. Associations were estimated using the Rao-Scott test, adjusted residual analysis, and simple and multiple logistic regression, calculating Odds Ratios (OR) and 95% confidence intervals (95%CI).

**Results:**

The prevalence of edentulism was 36.5%. A higher number of dentists per 3,500 inhabitants showed a protective effect (OR 0.96; p<0.01). The Brazilian Deprivation Index remained positively associated with the outcome, with the highest odds observed in the fifth quintile (OR 1.92; p<0.01). The absence of Dental Specialty Centers (OR 1.25; p=0.03) and Regional Dental Prosthesis Laboratories (OR 1.24; p=0.03) increased the prevalence of edentulism. Women (OR 1.24; p<0.01), age≥median of 69 years (OR 1.74; p<0.01), and low level of education (OR 4.25; p<0.01) were independent odds factors.

**Conclusion:**

Edentulism reflects social and structural inequalities in oral health. Expanding access to care and strengthening the public dental service network are essential measures to reduce inequities.

## INTRODUCTION

Edentulism remains an important public health issue, even in the face of advances observed in oral health care in recent decades. Defined as total tooth loss, it reflects social trajectories marked by accumulated inequalities throughout life. Its impacts extrapolate the functional dimension, affecting nutrition, communication, social participation and quality of life, as well as strengthening processes of vulnerability in more advanced ages^1^. It is a chronic, irreversible, and disabling condition. Eating or enjoying food and showing the teeth are among the most affected aspects in terms of quality of life of the edentulous population^2^.

Tooth loss significantly affects vulnerable people, resulting in both decreased oral functions and social silencing. In Brazil, among older adults, this condition reflects past policies focused on mutilating treatments for adults and prevention only aimed at schoolchildren, leading to exclusion and erasure of smiles in the older adult population^3^.

In Latin American countries, such as El Salvador and Colombia, population aging has significantly impacted the oral health of older people. Tooth loss and edentulism are associated with socioeconomic, behavioral, and functional determinants, as well as access to oral health services, which negatively affects quality of life, nutritional status, and functional capacity, especially among older adults with greater social vulnerability^4^.

In Brazil, national epidemiological surveys have been creating a basis for monitoring and assessing oral health conditions since 1986. With the National Survey of Oral Health (*Pesquisa Nacional de Saúde Bucal* – SB Brasil), these studies show that the prevalence of edentulism among older adults aged 65–74 years remains high^5^. This scenario contrasts with the goal proposed by the World Health Organization (WHO) for the year 2000, of maintaining at least 20 functional teeth in this age group. After more than two decades, a large part of the older adult population continues to live with the negative impacts of tooth loss on health^6^.

According to the available scientific evidence, the characteristics related to active aging have been seen as determinants of quality of life among older people^7^. The WHO’s theoretical framework for the promotion of active aging comprises three pillars: participation, health, and security^8^. Therefore, it is necessary to include health, in a comprehensive way, in the discussions of different government agendas, such as work and social security, as health conditions are associated with early retirement and worse capacity for work^9^.

Ferreira et al.^10^ analyzed edentulism in older adults in the national surveys SB Brasil of 2003, 2010, and 2023. Prevalence remained stable between 2003 and 2010, but fell significantly to 36.48% in 2023 in all regions and demographic groups. Reductions were evident, especially among older adults with lower levels of education, although inequalities related to race/skin color and education persist.

In this context, the present study is distinguished by the unprecedented character in articulating the microdata of SB Brasil 2023 with contextual indicators of social inequality—the Brazilian Deprivation Index (*Índice Brasileiro de Privação* – IBP) and the Social Vulnerability Index (*Índice de Vulnerabilidade Social* – IVS)—through multilevel modeling applied to the older adult population. This approach allows us to advance beyond descriptive and individual analyses traditionally employed in national surveys, while simultaneously capturing the effects of personal characteristics and territorial contexts on tooth loss. The incorporation of synthetic indicators of deprivation and vulnerability increases the explanatory capacity of the models, allowing us to observe how structural inequalities shape oral health outcomes in aging. From the methodological point of view, the study contributes by empirically operationalizing the social determination of oral health at different hierarchical levels, providing more refined evidence for the understanding of persistent inequalities and for the improvement of public policies on equity in care for older adults.

The social determination of oral health requires methods that allow empirical observation of its theoretical assumptions. Thus, in the present study, we aimed at analyzing contextual and individual factors associated with tooth loss among older people in Brazil.

## METHODS

A cross-sectional analysis was carried out with secondary data on the age group of 65–74 years, part of the population-based National Survey of Oral Health (SB Brasil), coordinated by the Ministry of Health. Data collection involved interviews and clinical examinations conducted by oral health professionals in the households, and field teams were trained and calibrated online and with the use of photographs on the Moodle^®^ platform, due to the context of the COVID-19 pandemic (in-lux method). Adequate coefficients of agreement were obtained (Kappa≥0.61). The sample was drawn and stratified by one or two-stage clusters (census tracts), and the collection was carried out between 2022 and 2024. With different geographic domains involved, the research covered all states and capitals. The sample design allowed for estimates for each of the five Brazilian regions. The survey followed the WHO standards for epidemiological surveys in oral health. The project was submitted to the National Research Ethics Committee (*Comissão Nacional de Ética na Pesquisa* – Conep), and was approved in 2021 under the Certificate of Presentation for Ethical Consideration (CAAE) No. 34497120.6.3001.0008 and Opinion No. 4.823.054^5,11,12,13,14,15^.

The dependent variable was the prevalence of edentulism, characterized by the dentate or edentulous (absence of all teeth) options. Although this definition is clear, objective, and widely used in epidemiological surveys, it excessively simplifies a phenomenon that has been continuously and heterogeneously manifested throughout the course of life. The dichotomization disregards severe, but not total, tooth losses that are clinically and functionally relevant, especially regarding chewing, phonetics, aesthetics, social interaction, and quality of life of older adults. It is known, therefore, that the choice for total edentulism as an outcome represents a conservative and extreme snippet of tooth loss, suitable for comparison with previous studies and the guarantee of analytical robustness, but which imposes limits on the interpretation of the results.

Independent variables were organized in two levels of analysis: individual variables (socioeconomic and demographic characteristics [sex, age, race/skin color, people per room, recipient of *Bolsa Família*—income transfer program of the Brazilian government—, and level of education]). Finally, the contextual variables related to socioeconomic status and the characteristics of the oral health network of the municipalities were analyzed, namely: number of dentists per 3,500 inhabitants in 2025, deemed as quantitative variable; and presence of Dental Specialty Centers (*Centros de Especialidades Odontológicas* – CEO) or Regional Dental Prosthesis Laboratories (*Laboratórios Regionais de Prótese Dentária* – LRPD) in 2025, deemed as dichotomous qualitative variable (yes or no).

These variables were based on the National Registry of Health Establishments (*Cadastro Nacional de Estabelecimentos de Saúde* – CNES). IVS 2010 (categorized as:

1. Very high vulnerability;
2. High vulnerability;
3. Average vulnerability;
4. Low vulnerability;
5. Very low vulnerability, source: Institute for Applied Economic Research – IPEA)^16^ and IBP 2010 (categorized in quintiles, source: Oswaldo Cruz Foundation, Center for Data and Knowledge Integration for Health – Fiocruz CIDACS)^17^ were processed as ordinal qualitative variables.

The variables were linked to the individual data through the code of the participant’s municipality of residence, according to the identification present in the database of SB Brasil 2023.

Individual and contextual independent variables were organized in blocks in the distal-to-proximal sequence in relation to the outcome (more distal contextual block, followed by the individual socioeconomic and demographic blocks, use of dental services, and impacts of oral health on daily activities). The hierarchization of the blocks is based on the theoretical conception proposed by Victora et al^18^. This structuralist-based conception of social processes guided the hierarchical modeling process, working both for the control of confounding variables and for the positioning of each block according to its degree of proximity to the outcome. Meanwhile, multilevel analysis, often known as hierarchical analysis, disregards the order in the modeling blocks, but has two functions: first, it enables the partitioning of variances in situations of complex samples in which the sample units are not independent, but clustered according to levels of geographic nature; second, it allows the inclusion of ecological/contextual variables, such as variables in addition to the individual level, allowing to verify the effect of the municipal level on the analyzed outcome^19^. The Rao-Scott^20^ test, an equivalent to the χ^2^ test for complex samples, was used to compare the prevalence of edentulism and its 95% confidence intervals (95%CI) between the different groups and with a 5% significance level. Furthermore, the analysis of standardized residuals was carried out for the association between the pairs of categories of the dependent variable and the independent variables. This analysis allows for the comparison of each category. The residuals (standardized difference between the observed and the expected counts) resulted in excess or lack of occurrence, being considered the values with positive excess count greater than 1.96, with a one-tailed 2.5% significance level, as it is only the observation of excesses. The Statistical Package for the Social Sciences (SPSS) 22 program was used for this analysis.

Subsequently, simple and multiple logistic regression analysis was performed using Odds Ratio (OR) as an effect measure. Mixed models of multilevel analysis were estimated, with random interception, considering the examined older individuals as the first level and the selected cities as the second level of analysis. In addition, a third level of analysis was included, the Federative Units, only for strengthening the partitioning of variances to better estimate the standard errors, aiming at the best accuracy of the confidence intervals. No state-level variables were used.

The analysis of association with Rao-Scott considered the complex design of the sample with its weights, strata, and sampling units available in the very SB Brasil database, using the SPSS complex sample method. In turn, in the multilevel model, individual weights were applied at the individual level, while contextual weights were considered at the aggregation levels. In these models, strata are considered as contextual levels. The hierarchical structure of the model allowed us to capture the intracluster correlation inherent in the sample design, making explicit specification of strata unnecessary. A hierarchical modeling process was conducted, considering the distance of each block to the outcome for adjustment. The variables that remained in each block were considered adjustment factors for subsequent blocks.

It is worth highlighting the distinction between blocks and levels. On the one hand, the blocks concern the positioning of the variables in relation to the edentulism outcome. In this sense, a hierarchical analysis by distal, medial, and proximal blocks to the outcome was used, regardless of the multilevel structure of the database. On the other hand, the levels refer to the nesting of the sample units within certain clusters, which prevents the assumption of sampling independence for the classical regression models. In this sense, the older adults are nested according to the levels of Federative Units and municipalities, not configuring independent units of analysis. Once again, a multilevel analysis can be conducted regardless of the definition of hierarchical blocks. In this study, a multilevel and hierarchical analysis was chosen, considering the aforementioned levels and the thematic positioning of each variable by blocks. The entry criterion in the multiple model was 25%; and for permanence, 5%. The order of entry of the variables was also weighted by the theoretical model together with the p-values. The MLwiN 3.05 program was used for this analysis.

The SB Brasil 2023 Project was approved by the National Research Ethics Committee (Opinion No. 4.823.054). Participation in the study was voluntary, by signing an Informed Consent Form.

### Data Availability Statement

The entire dataset that supports the results of this study is available upon request to the corresponding author. The dataset is not publicly available due to the integration of databases created by the authors.

## RESULTS

The prevalence of edentulism was 36.5% (95%CI 33.4–39.6%). We analyzed 9,745 older adults distributed in 27 states and 368 municipalities. We observed that the effective sample size (n_e_), calculated based on the variability of sample weights, was substantially lower than the nominal sample size (1,573 x 9,745). Consequently, the estimates show greater variance than those obtained from simple random sampling, reinforcing the need for analytical methods that consider the sample design. According to the results, there are significant differences in the dental conditions of the population, reflecting social inequalities and the pattern of use of oral health services. As shown in Table 1, the distribution of edentulous population proportions varied according to contextual, socioeconomic, and demographic factors.

**Table 1:** Distribution of dentate and edentulous participants according to contextual, socioeconomic, health service utilization, and self-rated oral health variables. Brazil, 2025.

Variable	Dentate	Edentulous	Total
	% (95%CI)	% (95%CI)	% (95%CI)
**Block 1 – Contextual**			
Social Vulnerability Index*			
Very high	3.5 (2.0–6.0)	6.6^†^ (3.4–12.5)	4.6 (2.7–7.9)
High	4.5 (3.2–6.4)	9.4^†^ (6.64–13.1)	7.1 (5.7–10.1)
Average	26.5(20.9–33.01)	29.6 (23–37.1)	27.6 (22–34.1)
Low	52.2^†^ (45.3–59.6)	42.2 (35–49.6)	48.7 (42.1–55.4)
Very low	10.3 (7–15)	12.3 (7.8–18.8)	11.0 (7.6–15.9)
Brazilian Deprivation Index quintiles*			
1st quintile	31.3^†^ (23.8–39.9)	20.7 (15.2–27.6)	27.4 (21.2–34.7)
2nd quintile	16.6 (12.4–21.8)	19.8 (14.4–26.7)	17.8 (13.6–22.8)
3rd quintile	19.4 (14.3–25.6)	18.6 (12.8–26.1)	19.1 (15.1–23.2)
4th quintile	18.3 (14.5–22,)	19.7 (15.2–25.2)	18.8 (14.0–23.9)
5th quintile	14.5 (11.4–18.1)	21.2^†^ (16.5–26.8)	16.9 (13.9–20.8)
Presence of CEO*			
No	24.1 (18.1 – 31.4)	31.0^†^ (23.6 – 20.6)	26.6 (20.6 – 33.7)
Yes	75.9^†^ (68.6 – 81.9)	69.0 (60.4 – 76.4)	73.4 (79.4– 50.4)
Presence of LRPD*			
No	29.5 (23.2 – 36.8)	36.4^†^ (28.7 – 44.9)	32.0 (25.7– 39.2)
Yes	70.5^†^ (63.2– 76.8)	63.6 (55.1 – 71.3)	68 (60.8– 74.3)
**Individual Block 2 – Sociodemographic**			
Sex*			
Women	58.5 (55.3–61.7)	63.8^†^ (60.2–67.2)	60.4 (58.2–62.7)
Men	41.5^†^ (38.3 – 44.7)	36.2 (32.8–39.8)	39.6 (37.3–41.8)
Age*			
Less than/equal to the median (69)	60.5^†^ (56.4 – 64.3)	48.5 (43.3–53.7)	56.1 (52.4–59.1)
Above the median (69)	39.5 (35.7–43.6)	51.5^†^ (46.3–56.7)	43.9 (47.6–43.9)
Race/skin color			
White	51 (46.8–55.1)	46.9 (41.9–52)	49 (45.8–53.2)
Black	47.3 (43.1–51.4)	52.0 (46.9–57)	49 (45.3–52.7)
Asian/Indigenous	1.8 (0.9–3.3)	1.1 (0.6–1.8)	1.5 (0.9–2.5)
People per room			
Less than/equal to the median (1)	57 (52.2–61.9)	59.3 (53.9–64.5)	57.9 (54.4–61.3)
Above the median (1)	42.9 (38.1–47.8)	40.7 (35.5–46.1)	42.1 (38.7–45.6)
Level of Education*			
No formal education	8.1 (3.0–6.5)	17.1^†^ (14.2–20.3)	11.4 (9.7–13.3)
Literate	4.4 (5.6–12.3)	7.8^†^ (4.3–13.7)	5.6 (3.6–8.8)
Elementary School	50.3 (46.7–53.2)	58.5^†^ (52.5–64.3)	53.1 (50.0–56.1)
High School	24.7^†^ (21.6–28.1)	13.7 (11.1–16.8)	20.7 (18.2–23.5)
Higher Education	12.8^†^ (10.3–15.7)	5.0 (4.1–6.0)	20.7 (18.5–23.5)
**Individual Block 3 – Use of health services**			
Have sought some dental service in the last year*			
Did not seek	42.8 (39.1–46.8)	71.2^†^ (67.0–75.3)	53.1 (49.4–56.8)
Have sought and was not seen	10.9^†^ (9.1–13.1)	6.7 (5.0–9.0)	9.4 (7.9–11.1)
Have sought and was scheduled for another day/place	8.6^†^ (6.6–11.0)	5.4 (3.5–8.2)	7.4 (5.9–9.3)
Have sought and was seen	37.7 (33.3–42.3)	16.6 (13.6–20.2)	30.0 (26.5–33.8)
Have some private/company/government agency dental insurance plan*			
Yes	9.9^†^ (7.6–12.8)	4.5 (2.7–7.3)	7.9 (6.1–10.3)
No	90.1 (87.2–92.4)	95.5^†^ (92.7–97.3)	92.1 (89.7–93.9)

*Rao-Scott test p-value<0.05;

^†^Standardized residuals>1.96.

Source: Prepared by the authors, 2025.

In the block of contextual variables, we observed that the prevalence of edentulism was higher among individuals living in areas with a very high and high social vulnerability index (adjusted residual greater than 1.96), while the lowest proportions were verified among those with average, low, and very low vulnerability. Regarding IBP, the proportions of edentulous individuals ranged from 20.7% in the first quintile to 21.2% in the fifth quintile. Municipalities in the highest deprivation quintile had a higher proportion of edentulous individuals. Among the municipalities without the presence of CEO or LRPD, there was a higher proportion of edentulous people.

In the block of socioeconomic and demographic variables, women represented 63.8% of the edentulous population. Among individuals aged above the median (69 years), 51.5% were edentulous. Regarding race/skin color, 52% of edentulous individuals were Black, 46.9% were white, and 1.1% were Asian or Indigenous. For the variable “number of people per room,” 59.3% of the edentulous individuals were in the group below or equal to the median of one person. As for level of education, there was a higher prevalence of edentulous people without formal education as well as between those who were literate and had an Elementary School degree.

In the block concerning health services, individuals who did not seek dental care in the last year represented 71.2% of the edentulous population. Those who sought care and were seen corresponded to 16.6% of edentulous individuals. Among those who had dental insurance plans, 4.5% were edentulous, while among those without insurance plans, 95.5% presented total tooth loss.

According to the null model with random intercept, there was significant variance in both levels, with emphasis on the municipal level (0.378 in the municipal versus 0.056 in the state). This result was expected, considering the largest number of sampling units at the municipal level. The intraclass correlation coefficient of the municipality was 10.2% and that of the state, 1.5%. Significant associations between the study outcome and demographic, socioeconomic, behavioral, and structural variables of oral health were found in simple and multiple analyses, as highlighted in Table 2. We observed that the increase in the number of dentists per inhabitant showed a protective effect, reducing the odds of the outcome (OR 0.96; p<0.01), while the highest social vulnerability (very high IVS) was associated with increased odds (OR 1.68; p=0.03). IBP maintained a statistically significant association even after adjustment, indicating a gradient of higher odds as socioeconomic conditions worsen, especially the last quintile (OR 1.92; p<0.01).

**Table 2:** Effect of contextual, socioeconomic variables, use of health services, and impacts of oral health on daily activities with edentulism, according to simple and multiple logistic regression model. Brazil, 2025.

Variable	Simple				Multiple			
	OR	95%CI		p-value	OR	95%CI		p-value
**Block 1 – Contextual**										
No. of dentists/inhab.	0.96	0.94	0.97	<0.01				
Social Vulnerability Index										
Very high	1.68	1.05	2.69	0.03				
High	1.36	0.9	2.06	0.14				
Average	1.19	0.82	1.74	0.36				
Low	1.07	0.75	1.52	0.72				
Very low	1							
Brazilian Deprivation Index quintiles										
5th quintile	2.25	1.6	3.15	<0.01	1.92	1.34	2.75	<0.01
4th quintile	1.61	1.15	2.24	<0.01	1.48	1.06	2.07	0.02
3rd quintile	1.3	0.92	1.83	0.13	1.24	0.88	1.73	0.21
2nd quintile	1.75	1.18	2.59	<0.01	1.72	1.17	2.53	<0.01
1st quintile	1				1			
Presence of CEO										
Yes	1				1			
No	1.45	1.20	1.75	<0.01	1.25	1.02	1.54	0.03
Presence of LRPD										
Yes	1							
No	1.24	1.02	1.51	0.03				
**Block 2 – Socioeconomic and demographic**										
Sex										
Women	1.17	1.07	1.27	<0.01	1.24	1.13	1.37	<0.01
Men	1				1			
Age										
Above the median	1.76	1.62	1.91	<0.01	1.74	1.59	1.90	<0.01
Below the median	1				1			
Race/skin color										
White	1							
Asian/Indigenous	0.73	0.53	1.01	0.06				
Black	1.06	0.97	1.17	0.20				
People per room										
Less than/equal to the median (1)	1				1			
Above the median (1)	1.14	1.05	1.24	<0.01	1.11	1.02	1.22	<0.01
*Bolsa Família* recipient										
No	1							
Yes	1.23	1.08	1.4	<0.01				
Level of Education										
No formal education	4.61	3.78	5.62	<0.01	4.25	3.45	5.25	<0.01
Literate	3.70	2.86	4.8	<0.01	3.35	2.53	4.42	<0.01
Elementary School	2.59	2.18	3.06	<0.01	2.59	2.16	3.10	<0.01
High School	1.62	1.35	1.94	<0.01	1.65	1.36	2.00	<0.01
Higher Education	1				1			
**Block 3 – Use of health services**										
Have sought a dentist										
Have never sought	3.12	2.81	3.46	<0.01	2.72	2.42	3.05	<0.01
Have sought, but was not seen	1.49	1.26	1.77	<0.01	1.32	1.09	1.58	<0.01
Have sought and was scheduled	1.1	0.89	1.37	0.36	1.12	0.88	1.41	0.36
Have sought and was seen	1				1			
Type of service used										
Private or insurance plan	1							
Have never sought it	3.11	2.78	3.48	<0.01				
Public	1.25	1.08	1.44	<0.01				
Dental insurance plan										
Yes	1				1.00			
No	2.53	2.13	3.00	<0.01	1.87	1.53	2.29	<0.01

p<0.05 values were considered statistically significant.

OR: Odds Ratio; 95%CI: 95% confidence interval; LRPD: Regional Dental Prosthesis Laboratories; CEO: Dental Specialty Centers.

Source: Prepared by the authors, 2025.

The absence of CEO was also associated with a higher odds of the outcome in the multiple analysis (OR 1.25; p=0.03), stressing the importance of the care structure in reducing inequalities. The type of service used (private, insurance plan, or public) and the history of seeking dental care influenced the odds of the outcome, and individuals who had never sought the service presented a higher odds of the event (OR 2.72; p<0.01). Older people without dental insurance plan had 87% higher odds of edentulism (OR 1.87; p<0.01).

Among the individual characteristics, women (OR 1.24; p<0.01) and age above the median (≥69 years; OR 1.74; p<0.01) were independent odds factors. Level of education proved to be the determinant with the highest effect size, with a progressive increase in odds among individuals with lower levels of education; those without formal education presented four-time higher odds than those with a Higher Education degree (OR 4.25; p<0.01).

## DISCUSSION

Although national surveys of oral health show a trend of greater dental preservation among older adults^21^, this advance has not equitably occurred. The persistence of differences according to level of education and race/skin color suggests that possible population gains coexist with structural inequalities between subgroups, mediated by socioeconomic and territorial factors^22,23^.

Tooth loss is a complex phenomenon, resulting from the interaction between social, environmental, clinical, and behavioral factors^24^, with repercussions that extrapolate the oral cavity^25^. Authors of previous studies using data from SB Brasil 2003 have already demonstrated the effect of access to dental services and the standard of care on tooth loss^26^. From the perspective of functional dentition^27^, inequalities persist according to socioeconomic status, showing that the maintenance of 20 or more natural teeth remains socially distributed^28^.

Consistently, the higher availability of dentists per inhabitant showed a protective effect on the adjusted model, corroborating that dental preservation depends not only on individual characteristics, but also on the organization of care provision. Thus, tooth loss in aging must be understood in the context of social conditions and the structure of care systems.

The non-permanence of the race/skin color variable in the adjusted model may reflect mediation by socioeconomic and territorial factors, and should not be interpreted as absence of racial inequality. Nonetheless, the sample robustness of SB Brasil 2023 strengthens the legitimacy of the findings.

Social inequality is portrayed in the expression of the smile. According to a report produced by the Brazilian Monitoring Center of Inequalities (*Observatório Brasileiro das Desigualdades*), the significant deficit of public services falls upon the poorest, affecting the most basic conditions of citizenship at all stages of life^29^.

In this context, analyzing tooth loss among older adults is an important indicator of the functioning of the oral health system of a country. According to projections for Brazil, edentulism will affect 85.96% of older adults in 2040, which represents more than 64 million people^21^.

Fagundes et al.^30^ highlight the importance of understanding health macrodeterminants to combat disparities and promote equity. The authors analyzed the distribution of edentulism in five dimensions between 2000, 2010, and 2019 in 204 countries and territories. Among people aged 55 years or older, there was an increase in the rates of prevalence, incidence, and years lived with disability related to edentulism in countries with high Gross Domestic Product (GDP) from 2010 to 2019, with persistent inequalities. Countries with greater economic development, better governance, and social policies presented higher rates of edentulism, possibly due to the dominant model of individualized care to oral health.

Tackling edentulism represents a challenge of significant magnitude, requiring a comprehensive approach from public policies, focused both on preventive perspectives and on aspects for ensuring access to rehabilitating treatment. Among the guidelines for the National Oral Health Policy, known as *Brasil Sorridente* [Smiling Brazil], in 2004, these aspects were included by procedures for partial or total recovery of the lost capacities as a result of the disease and reintegration of the individual to their social environment and professional activity^5,31^. By implementing the National Oral Health Policy within the Brazilian Unified Health System (SUS), as enforced by Law No. 14,572, of 2023, oral health was included in the scope of action of the SUS, thus operationalizing initiatives aimed at the social production of oral health and, specifically, at dental initiatives at all levels of care^32^. Despite the provision of dental prostheses in the SUS, access to this service is restricted. Public resources and the LRPD are limited to performing total prostheses, with a capacity lower than the needs of the population^33^.

Although the cross-sectional design limits causal inferences, the multilevel analysis allowed us to investigate the articulation between individual and contextual factors. However, other limitations can be observed, among them potential multicollinearity between contextual variables.

Losing teeth while aging is unnatural^34^. The results presented in this study illustrate an avoidable, unfair, and unnecessary panorama of social determination of oral health. Ensuring the overcoming of weaknesses in the oral health care network that hinder the maintenance of teeth in the oral cavity is a priority, so that no smile is hidden.

“The silence of lost smiles” reflects, in an analytical way, how tooth loss among older people in Brazil symbolizes historical social inequalities. The smile becomes a marker of the exclusion from health care and state omission, aggravated by care models that generated mutilation throughout generations. Although there are advances in dental preservation, according to SB Brasil 2023, disparities related to race/skin color and level of education persist. Edentulism is the result of avoidable political and structural choices, and overcoming it requires strengthening comprehensive and equitable access to oral health, recognizing the smile as a fundamental human right.
