# Supplementary material for: The silence of lost smiles: multilevel analysis of edentulism among older adults in contextual and individual interfaces in Brazil (SB Brasil 2023)
Source: Rev Bras Epidemiol. 2026 Jul 27;29(Suppl 1):e260028supl1. doi: 10.1590/1980-549720260028.supl.1 (PMC13405552; doi:10.1590/1980-549720260028.supl.1)
Supplement: Translation

# Translation: O silêncio dos sorrisos perdidos: análise multinível do edentulismo em pessoas idosas nas interfaces contextuais e individuais no Brasil (SB Brasil 2023)

**DOI:** 10.1590/1980-549720260028.supl.1.2

**Authors:** Rafael da Silveira Moreira, Camila Caroline da Silva, Lucas Fernando Rodrigues dos Santos, Tainá Faustino Mafra, Herika de Arruda Mauricio

**Affiliations:** I Fundação Oswaldo Cruz, Instituto Aggeu Magalhães, Department of Public Health – Recife (PE), Brasil.; II Universidade Federal de Pernambuco, School of Medicine – Recife (PE), Brasil.; III Universidade de Pernambuco, School of Dentistry – Recife (PE), Brasil.

**Keywords:** Perda de dente, Idoso, Odontologia em saúde pública, Saúde bucal, Inquéritos de saúde bucal

## Abstract

**Objetivo:**

Analisar os fatores contextuais e individuais associados ao edentulismo em pessoas idosas de 65 a 74 anos no Brasil, inquérito nacional SB Brasil 2023.

**Métodos:**

Estudo transversal com 9.745 participantes distribuídos nas 27 Unidades Federativas. A coleta de dados utilizou questionários e exames clínicos padronizados. Foram analisadas variáveis contextuais dos municípios e variáveis individuais demográficas, socioeconômicas, de uso dos serviços e de autopercepção em saúde bucal. As associações foram estimadas pelo teste de Rao-Scott, análise de resíduos ajustados e regressão logística simples e múltipla, calculando-se *Odds Ratios* (OR) e intervalos de confiança de 95% (IC95%).

**Resultados:**

A prevalência de edentulismo foi de 36,5%. O maior número de cirurgiões-dentistas a cada 3.500 habitantes mostrou efeito protetor (OR 0,96; p<0,001). O Índice Brasileiro de Privação manteve associação positiva com o desfecho, com maior chance no quinto quintil (OR 1,92; p<0,01). A ausência de Centros de Especialidades Odontológicas (OR 1,25; p=0,03) e de Laboratórios Regionais de Prótese Dentária (OR 1,24; p=0,03) aumentou a prevalência do edentulismo. Sexo feminino (OR 1,24; p<0,01), idade ≥ mediana de 69 anos (OR 1,74; p<0,01) e baixa escolaridade (OR 4,25; p<0,01) foram fatores de maior chance independentes.

**Conclusão:**

O edentulismo expressa desigualdades sociais e estruturais em saúde bucal. Ampliar a assistência e fortalecer a rede pública são medidas essenciais para reduzir as iniquidades.

## INTRODUÇÃO

O edentulismo permanece um importante problema de saúde pública, mesmo diante dos avanços observados na atenção à saúde bucal nas últimas décadas. Definido pela perda dentária total, constitui a expressão de trajetórias sociais marcadas por desigualdades acumuladas ao longo da vida. Seus impactos extrapolam a dimensão funcional, repercutindo na alimentação, na comunicação, na participação social e na qualidade de vida, além de reforçar processos de vulnerabilização em idades mais avançadas^1^. Trata-se de uma condição crônica, irreversível e incapacitante. Comer ou apreciar a comida e mostrar os dentes estão entre os aspectos mais afetados em termos de qualidade de vida da população edêntula^2^.

A perda dentária afeta de forma marcante pessoas vulneráveis, resultando tanto na diminuição das funções bucais quanto no silenciamento social. No Brasil, entre idosos, essa condição reflete políticas passadas focadas em tratamentos mutiladores para adultos e em prevenção apenas voltada a escolares, levando à exclusão e ao apagamento de sorrisos na população idosa^3^.

Em países latino-americanos como El Salvador e Colômbia, o envelhecimento populacional tem impactado significativamente a saúde bucal das pessoas idosas. A perda dentária e o edentulismo estão associados a determinantes socioeconômicos, comportamentais e funcionais, bem como ao acesso aos serviços de saúde bucal, o que repercute negativamente na qualidade de vida, no estado nutricional e na capacidade funcional, especialmente entre idosos em situação de maior vulnerabilidade social^4^.

No Brasil, inquéritos epidemiológicos nacionais vêm desde 1986 construindo uma base de monitoramento e de avaliação da condição de saúde bucal. Com a Pesquisa Nacional de Saúde Bucal (SB Brasil), tais estudos mostram que a prevalência do edentulismo entre pessoas idosas de 65 a 74 anos permanece elevada^5^. Esse cenário contrasta com a meta proposta pela Organização Mundial da Saúde (OMS) para o ano 2000, de pelo menos 20 dentes funcionais nessa faixa etária. Passadas mais de duas décadas, grande parte da população idosa continua a conviver com os impactos negativos da perda dentária sobre a saúde^6^.

As evidências científicas disponíveis demonstram que as características ligadas ao envelhecimento ativo têm sido vistas como determinantes da qualidade de vida entre pessoas idosas^7^. O marco teórico da OMS para a promoção do envelhecimento ativo compreende três pilares: participação, saúde e segurança^8^. Por isso, é preciso incluir a saúde de forma transversal nas discussões das diferentes pastas governamentais, tais como trabalho e previdência, uma vez que as condições de saúde estão associadas à aposentadoria precoce e à pior capacidade para o trabalho^9^.

Ferreira et al.^10^ analisaram o edentulismo em idosos nos levantamentos nacionais SB Brasil de 2003, 2010 e 2023. A prevalência permaneceu estável entre 2003 e 2010, mas caiu significativamente para 36,48% em 2023 em todas as regiões e grupos demográficos. Reduções foram evidentes, especialmente entre idosos com menor escolaridade, embora desigualdades relacionadas à raça/cor da pele e à escolaridade persistam.

Nesse contexto, o presente estudo se distingue pelo caráter inédito ao articular os microdados do SB Brasil 2023 com indicadores contextuais de desigualdade social — o Índice Brasileiro de Privação (IBP) e o Índice de Vulnerabilidade Social (IVS) — por meio de modelagem multinível aplicada à população idosa. Tal abordagem permite avançar para além das análises descritivas e individuais tradicionalmente empregadas nos inquéritos nacionais, ao captar simultaneamente os efeitos das características pessoais e dos contextos territoriais na perda dentária. A incorporação de indicadores sintéticos de privação e vulnerabilidade amplia a capacidade explicativa dos modelos, possibilitando observar como desigualdades estruturais moldam os desfechos em saúde bucal no envelhecimento. Do ponto de vista metodológico, o estudo contribui ao operacionalizar empiricamente a determinação social da saúde bucal em diferentes níveis hierárquicos, oferecendo evidências mais refinadas para a compreensão das iniquidades persistentes e para o aprimoramento de políticas públicas orientadas à equidade no cuidado à pessoa idosa.

A determinação social da saúde bucal exige métodos que permitam a observação empírica de seus pressupostos teóricos. Assim, o objetivo do estudo é analisar fatores contextuais e individuais associados à perda dentária entre pessoas idosas no Brasil.

## MÉTODOS

Realizou-se uma análise transversal com dados secundários relacionados à faixa etária de 65 a 74 anos integrante do Inquérito Nacional de Saúde Bucal de base populacional de 2023 (SB Brasil), coordenado pelo Ministério da Saúde. A coleta de dados envolveu entrevistas e exames bucais conduzidos por profissionais de saúde bucal nos domicílios, sendo as equipes de campo treinadas e calibradas por meio de abordagem *on-line* e com o uso de fotografias na plataforma Moodle^®^, por conta do contexto de pandemia da COVID-19, método esse conhecido por *in-lux*. Foram obtidos adequados coeficientes de concordância (Kappa≥0,61). A amostra foi sorteada e estratificada por conglomerados (setores censitários) em um ou dois estágios, e a coleta foi realizada entre os anos de 2022 e 2024. Com diferentes domínios geográficos envolvidos, a pesquisa abrangeu todos os estados e capitais. O delineamento amostral permitiu estimativas para cada uma das cinco regiões brasileiras. O inquérito seguiu as normas da OMS para a realização de levantamentos epidemiológicos em saúde bucal. O projeto foi submetido à apreciação da Comissão Nacional de Ética na Pesquisa (Conep), sendo aprovado em 2021 sob o Certificado de Apresentação para Apreciação Ética (CAAE) 34497120.6.3001.0008 e parecer número 4.823.054^5,11,12,13,14,15^.

A variável dependente foi a prevalência de edentulismo, caracterizada pelas opções dentado ou edêntulo (ausência de todos os dentes). Embora essa definição seja clara, objetiva e amplamente utilizada em inquéritos epidemiológicos, ela simplifica excessivamente um fenômeno que se manifesta de forma contínua e heterogênea ao longo do curso da vida. A dicotomização desconsidera perdas dentárias severas, porém não totais, que são clinicamente e funcionalmente relevantes, especialmente no que se refere à mastigação, fonética, estética, interação social e qualidade de vida das pessoas idosas. Reconhece-se, portanto, que a escolha pelo edentulismo total como desfecho representa um recorte conservador e extremo da perda dentária, adequado à comparabilidade com estudos prévios e à garantia de robustez analítica, mas que impõe limites à interpretação dos resultados.

Foram utilizadas variáveis independentes organizadas em dois níveis de análise: as de ordem individual (características socioeconômicas e demográficas [sexo, idade, raça/cor, pessoas por cômodo, bolsa família e escolaridade]). Por fim, foram analisadas as variáveis de ordem contextual, referentes à condição socioeconômica e às características da rede odontológica dos municípios: número de cirurgiões-dentistas a cada 3.500 habitantes no ano de 2025, tratada como variável quantitativa; e existência de Centros de Especialidades Odontológicas (CEO) ou de Laboratórios Regionais de Prótese Dentária (LRPD) em 2025, considerada como variável qualitativa dicotômica (possui ou não possui).

Essas variáveis tiveram como fonte o Cadastro Nacional de Estabelecimentos de Saúde (CNES). O IVS 2010 (categorizado como:

1. Muito alta vulnerabilidade;
2. Alta vulnerabilidade;
3. Média vulnerabilidade;
4. Baixa vulnerabilidade;
5. Muito baixa vulnerabilidade, fonte Instituto de Pesquisa Econômica Aplicada — IPEA)^16^ e o IBP 2010 (categorizado em quintis, fonte Fundação Oswaldo Cruz, Centro de Integração de Dados e Conhecimentos para Saúde — Fiocruz CIDACS)^17^ foram processados como qualitativas ordinais.

As variáveis foram vinculadas aos dados individuais por meio do código do município de residência do participante, conforme identificação presente no banco de dados do SB Brasil 2023.

As variáveis independentes individuais e contextuais foram organizadas em blocos na sequência distal para proximal ao desfecho (bloco contextual como mais distal, seguido pelos blocos individuais socioeconômico e demográfico, uso de serviços odontológicos e impactos da saúde bucal nas atividades diárias). A hierarquização dos blocos se baseia na concepção teórica proposta por Victora et al^18^. Essa concepção de raiz estruturalista dos processos sociais guiou o processo de modelagem hierarquizada, funcionando tanto para o controle de variáveis de confundimento quanto para o posicionamento de cada bloco segundo seu grau de proximidade ao desfecho. Entrementes, a análise multinível, muitas vezes conhecida como análise hierárquica, não disputa ordem nos blocos de modelagem, mas possui duas funções: primeiramente proporciona o particionamento das variâncias em situações de amostras complexas em que as unidades amostrais não são independentes, mas clusterizadas segundo níveis de pertencimentos geográficos; segundo, permite a inclusão de variáveis ecológicas/contextuais, como variáveis além do nível individual, permitindo verificar o efeito do nível municipal sobre o desfecho analisado^19^. O teste de Rao-Scott^20^, um equivalente ao teste do χ^2^ para amostras complexas, foi usado para comparar a prevalência de edentulismo e seus intervalos de confiança de 95% (IC95%) entre os diferentes grupos e com nível de significância de 5%. Adicionalmente foi realizada a análise de resíduos padronizados para a associação entre os pares de categorias da variável dependente e das independentes. Essa análise possibilita a comparação de cada categoria. Os resíduos (diferença padronizada entre as contagens observadas e esperadas) resultaram em excesso ou falta de ocorrência, sendo considerados os valores com contagem de excesso positivo superior a 1,96, com nível de significância de 2,5% unicaudal, por se tratar apenas da observação dos excessos. Para essa análise foi utilizado o programa Statistical Package for the Social Sciences (SPSS) 22.

Em seguida, foi realizada análise de regressão logística simples e múltipla utilizando *Odds Ratio* (OR) como medida de efeito. Foram estimados modelos mistos de análise multinível, com intercepto aleatório, considerando as pessoas idosas examinadas como o primeiro nível e as cidades selecionadas como o segundo nível da análise. Adicionalmente, foi incluído um terceiro nível de análise, as Unidades da Federação, apenas como reforço da partição das variâncias para melhor estimativa dos erros padrão, visando à melhor precisão dos intervalos de confiança. Não foram utilizadas variáveis do nível estadual.

As análises de associação com Rao-Scott consideraram o desenho complexo da amostra com seus pesos, estratos e unidades de amostragem disponíveis no próprio banco de dados do SB Brasil, usando o método de amostra complexa do SPSS. Já no modelo multinível, os pesos individuais foram aplicados no nível do indivíduo, enquanto os pesos contextuais foram considerados nos níveis agregados. Nesses modelos, os estratos entram como os níveis contextuais. A estrutura hierárquica do modelo permitiu capturar a correlação intracluster inerente ao desenho amostral, tornando desnecessária a especificação explícita de estratos. Foi conduzido um processo hierarquizado de modelagem, considerando-se a distância de cada bloco ao desfecho para o ajuste. As variáveis que permaneceram em cada bloco foram consideradas fatores de ajuste para os blocos subsequentes.

Vale ressaltar a distinção entre blocos e níveis. Por um lado, os blocos dizem respeito ao posicionamento das variáveis em relação ao desfecho edentulismo. Nesse sentido, recorremos a uma análise hierarquizada por blocos distais, mediais e proximais ao desfecho, independente da estrutura multinível do banco de dados. Por outro lado, os níveis se referem ao aninhamento das unidades amostrais em determinados aglomerados, o que impede a suposição de independência amostral para os modelos clássicos de regressão. Nesse sentido, as pessoas idosas estão aninhadas segundo os níveis Unidades da Federação e municípios, não configurando unidades independentes de análise. Mais uma vez, uma análise apenas multinível pode ser conduzida independentemente da definição de blocos hierarquizados. Nesse estudo, optamos por uma análise multinível e hierarquizada, considerando os níveis já mencionados e o posicionamento temático de cada variável por blocos. O critério para a entrada no modelo múltiplo foi de 25% e de permanência foi de 5%. A ordem de entrada das variáveis também foi ponderada pelo modelo teórico em conjunto com os p-valores. Para essa análise foi utilizado o programa MLwiN 3.05.

O Projeto SB Brasil 2023 foi aprovado pelo Comitê Nacional de Ética em Pesquisa (Parecer n^o^ 4.823.054). A participação no estudo foi voluntária, mediante assinatura do Termo de Consentimento Livre e Esclarecido (TCLE).

### Declaração de disponibilidade de dados

Todo o conjunto de dados que dá suporte aos resultados deste estudo está disponível mediante solicitação ao autor correspondente. O conjunto de dados não está publicamente disponível devido à integração de bancos de dados realizados pelos autores.

## RESULTADOS

A prevalência do edentulismo foi de 36,5% (IC95% 33,4–39,6%). Foram analisadas 9.745 pessoas idosas distribuídas em 27 estados e 368 municípios. Observou-se que o tamanho efetivo da amostra (n_e_), calculado com base na variabilidade dos pesos amostrais, foi substancialmente inferior ao tamanho amostral nominal (1.573 x 9.745). Consequentemente, as estimativas apresentam maior variância do que aquelas obtidas sob amostragem aleatória simples, reforçando a necessidade de métodos analíticos que considerem o desenho da amostra. Os resultados revelam diferenças expressivas nas condições dentárias da população, refletindo desigualdades sociais e no padrão de utilização dos serviços de saúde bucal. Conforme demonstrado na Tabela 1, a distribuição das proporções de edêntulos variou de acordo com fatores contextuais, socioeconômicos e demográficos.

**Tabela 1:** Distribuição dos participantes dentados e edêntulos segundo variáveis contextuais, socioeconômicas, de serviços de saúde e de autopercepção em saúde bucal. Brasil, 2025.

Variável	Dentados	Edêntulos	Total
	% (IC95%)	% (IC95%)	% (IC95%)
**Bloco 1- Contextuais**			
Índice de Vulnerabilidade Social*			
Muito alta	3,5 (2,0–6,0)	6,6^†^ (3,4–12,5)	4,6 (2,7–7,9)
Alta	4,5 (3,2–6,4)	9,4^†^ (6,64–13,1)	7,1 (5,7–10,1)
Média	26,5(20,9–33,01)	29,6 (23–37,1)	27,6 (22–34,1)
Baixa	52,2^†^ (45,3–59,6)	42,2 (35–49,6)	48,7(42,1–55,4)
Muito baixa	10,3 (7–15)	12,3 (7,8–18,8)	11,0 (7,6–15,9)
Quintis Índice Brasileiro de Privação*			
Quintil 1	31,3^†^ (23,8–39,9)	20,7 (15,2–27,6)	27,4 (21,2–34,7)
Quintil 2	16,6 (12,4–21,8)	19,8 (14,4–26,7)	17,8 (13,6–22,8)
Quintil 3	19,4 (14,3–25,6)	18,6 (12,8–26,1)	19,1 (15,1–23,2)
Quintil 4	18,3 (14,5–22,)	19,7 (15,2–25,2)	18,8 (14,0–23,9)
Quintil 5	14,5 (11,4–18,1)	21,2^†^ (16,5–26,8)	16,9 (13,9–20,8)
Possui CEO*			
Não	24,1 (18,1 – 31,4)	31,0^†^ (23,6 –20,6)	26,6 (20,6 – 33,7)
Sim	75,9^†^ (68,6 – 81,9)	69,0 (60,4 – 76,4)	73,4 (79,4– 50,4)
Possui LRPD*			
Não	29,5 (23,2 – 36,8)	36,4^†^ (28,7 – 44,9)	32,0 (25,7– 39,2)
Sim	70,5^†^ (63,2– 76,8)	63,6 (55,1 – 71,3)	68 (60,8– 74,3)
**Bloco Individual 2- Sociodemográficas**			
Sexo*			
Feminino	58,5 (55,3–61,7)	63,8^†^ (60,2–67,2)	60,4 (58,2–62,7)
Masculino	41,5^†^ (38,3 – 44,7)	36,2 (32,8–39,8)	39,6 (37,3–41,8)
Idade*			
Menor/igual a mediana (69)	60,5^†^ (56,4 – 64,3)	48,5 (43,3–53,7)	56,1(52,4–59,1)
Acima da mediana (69)	39,5 (35,7–43,6)	51,5^†^ (46,3–56,7)	43,9 (47,6–43,9)
Raça/cor			
Branca	51 (46,8–55,1)	46,9 (41,9–52)	49 (45,8–53,2)
Negra	47,3 (43,1–51,4)	52,0 (46,9–57)	49 (45,3–52,7)
Amarela/Indígena	1,8 (0,9–3,3)	1,1 (0,6–1,8)	1,5 (0,9–2,5)
Pessoas por cômodo			
Menor/igual à mediana(1)	57 (52,2–61,9)	59,3 (53,9–64,5)	57,9 (54,4–61,3)
Acima da mediana (1)	42,9 (38,1–47,8)	40,7 (35,5–46,1)	42,1 (38,7–45,6)
Escolaridade*			
Sem escolaridade	8,1 (3,0–6,5)	17,1^†^ (14,2–20,3)	11,4 (9,7–13,3)
Alfabetização	4,4 (5,6–12,3)	7,8^†^ (4,3–13,7)	5,6 (3,6–8,8)
Fundamental	50,3 (46,7–53,2)	58,5^†^ (52,5–64,3)	53,1 (50,0–56,1)
Ensino médio	24,7^†^ (21,6–28,1)	13,7 (11,1–16,8)	20,7 (18,2–23,5)
Ensino superior	12,8^†^ (10,3–15,7)	5,0 (4,1–6,0)	20,7 (18,5–23,5)
**Bloco Individual 3- Uso dos serviços de saúde**			
No último ano, procurou algum serviço odontológico*			
Não procurou	42,8 (39,1–46,8)	71,2^†^ (67,0–75,3)	53,1 (49,4–56,8)
Procurou e não foi atendido	10,9^†^ (9,1–13,1)	6,7 (5,0–9,0)	9,4 (7,9–11,1)
Procurou e foi agendado para outro dia/local	8,6^†^ (6,6–11,0)	5,4 (3,5–8,2)	7,4 (5,9–9,3)
Procurou e foi atendido	37,7 (33,3–42,3)	16,6 (13,6–20,2)	30,0 (26,5–33,8)
Possui algum plano odontológico particular/empresa/órgão público*			
Sim	9,9^†^ (7,6–12,8)	4,5 (2,7–7,3)	7,9 (6,1–10,3)
Não	90,1 (87,2–92,4)	95,5^†^ (92,7–97,3)	92,1 (89,7–93,9)

*Teste Rao-Scott valor-p<0,05;

^†^Resíduos padronizados>1,96.

Fonte: Os autores; 2025.

No bloco de variáveis contextuais, observou-se que a prevalência de edentulismo foi maior entre os indivíduos residentes em áreas com índice de vulnerabilidade social muito alto e alto (resíduo ajustado maior que 1,96), enquanto as menores proporções ocorreram entre aqueles de média, baixa e muito baixa vulnerabilidade. Com relação ao IBP, as proporções de edêntulos variaram de 20,7% no primeiro quintil a 21,2% no quinto quintil. Municípios no maior quintil de privação apresentaram maior proporção de edêntulos. Entre os municípios sem a presença de CEO ou de LRPD, verificou-se maior proporção de edêntulos.

No bloco de variáveis socioeconômicas e demográficas, o sexo feminino representou 63,8% dos edêntulos. Entre os indivíduos com idade acima da mediana (69 anos), 51,5% eram edêntulos. Quanto à raça/cor, observaram-se 52% de edêntulos entre pessoas negras, 46,9% entre brancas e 1,1% entre amarelas ou indígenas. Para a variável número de pessoas por cômodo, 59,3% dos edêntulos encontravam-se no grupo abaixo ou igual à mediana de uma pessoa. Com relação à escolaridade, verificou-se maior prevalência de edêntulos sem escolaridade, assim como entre alfabetizados e com ensino fundamental.

No bloco referente aos serviços de saúde, os indivíduos que não procuraram atendimento odontológico no último ano representaram 71,2% dos edêntulos. Aqueles que procuraram e foram atendidos corresponderam a 16,6% de edêntulos. Entre os que possuíam plano odontológico, 4,5% eram edêntulos, enquanto, entre os sem plano, 95,5% apresentavam perda dentária total.

O modelo nulo com intercepto aleatório apresentou variância significativa em ambos os níveis, com destaque no nível municipal (0,378 no municipal contra 0,056 no estadual). Esse resultado já era esperado, haja vista o maior número de unidades amostrais no nível municipal. O Coeficiente de Correlação Intraclasse do município foi 10,2% e o do estado foi 1,5%. As análises simples e múltiplas (Tabela 2) sublinharam associações significativas entre o desfecho estudado e variáveis demográficas, socioeconômicas, comportamentais e estruturais de saúde bucal. Observou-se que o aumento do número de cirurgiões-dentistas por habitante apresentou efeito protetor, reduzindo a chance do desfecho (OR 0,96; p<0,01), enquanto a maior vulnerabilidade social (IVS muito alto) esteve associada a uma chance aumentada (OR 1,68; p=0,03). O IBP manteve associação estatisticamente significativa mesmo após ajuste, indicando um gradiente de maior chance à medida que pioram as condições socioeconômicas, com destaque para o último quintil (OR 1,92; p<0,01).

**Tabela 2:** Efeito das variáveis contextuais, socioeconômicas, uso de serviços de saúde e de impactos da saúde bucal nas atividades diárias com o edentulismo, segundo modelo de regressão logística simples e múltipla. Brasil, 2025.

Variável	Simples				Múltipla			
	OR	IC95%		Valor-p	OR	IC95%		Valor-p
**Bloco 1- Contextuais**										
Nº de CD /Hab.	0,96	0,94	0,97	<0,01				
Índice de Vulnerabilidade Social										
Muito alta	1,68	1,05	2,69	0,03				
Alta	1,36	0,9	2,06	0,14				
Média	1,19	0,82	1,74	0,36				
Baixa	1,07	0,75	1,52	0,72				
Muito baixa	1							
Quintis Índice Brasileiro de Privação										
Quintil 5	2,25	1,6	3,15	<0,01	1,92	1,34	2,75	<0,01
Quintil 4	1,61	1,15	2,24	<0,01	1,48	1,06	2,07	0,02
Quintil 3	1,3	0,92	1,83	0,13	1,24	0,88	1,73	0,21
Quintil 2	1,75	1,18	2,59	<0,01	1,72	1,17	2,53	<0,01
Quintil 1	1				1			
Possui CEO										
Sim	1				1			
Não	1,45	1,20	1,75	<0,01	1,25	1,02	1,54	0,03
Possui LRPD										
Sim	1							
Não	1,24	1,02	1,51	0,03				
**Bloco 2 - Socioeconômicas e demográficas**										
Sexo										
Feminino	1,17	1,07	1,27	<0,01	1,24	1,13	1,37	<0,01
Masculino	1				1			
Idade										
Acima da mediana	1,76	1,62	1,91	<0,01	1,74	1,59	1,90	<0,01
Abaixo da mediana	1				1			
Raça/cor										
Branca	1							
Amarelo/Indígena	0,73	0,53	1,01	0,06				
Negra	1,06	0,97	1,17	0,20				
Pessoas por cômodo										
Menor/igual mediana1	1				1			
Acima mediana 1	1,14	1,05	1,24	<0,01	1,11	1,02	1,22	<0,01
Bolsa Família										
Não	1							
Sim	1,23	1,08	1,4	<0,01				
Escolaridade										
Sem escolaridade	4,61	3,78	5,62	<0,01	4,25	3,45	5,25	<0,01
Alfabetização	3,70	2,86	4,8	<0,01	3,35	2,53	4,42	<0,01
Fundamental	2,59	2,18	3,06	<0,01	2,59	2,16	3,10	<0,01
Ensino médio	1,62	1,35	1,94	<0,01	1,65	1,36	2,00	<0,01
Ensino superior	1				1			
**Bloco 3- uso dos serviços de saúde**										
Procurou o Cirurgião-dentista										
Nunca procurou	3,12	2,81	3,46	<0,01	2,72	2,42	3,05	<0,01
Procurou, mas não foi atendido	1,49	1,26	1,77	<0,01	1,32	1,09	1,58	<0,01
Procurou e foi agendado	1,1	0,89	1,37	0,36	1,12	0,88	1,41	0,36
Procurou e foi atendido	1				1			
Tipo de uso do serviço										
Particular ou plano	1							
Nunca	3,11	2,78	3,48	<0,01				
Pública	1,25	1,08	1,44	<0,01				
Plano Odontológico										
Sim	1				1,00			
Não	2,53	2,13	3,00	<0,01	1,87	1,53	2,29	<0,01

Valores de p < 0,05 foram considerados estatisticamente significativos.

OR: odds ratio; IC95%: intervalo de confiança de 95%; LRPD: Laboratórios Regionais de Prótese Dentária; CEO: Centros de Especialidades Odontológicas.

Fonte: Os autores; 2025.

A ausência de CEO também se associou a uma maior chance do desfecho na análise múltipla (OR 1,25; p=0,03), reforçando a importância da estrutura assistencial na redução das iniquidades. O tipo de serviço procurado (particular, plano ou público) e o histórico de busca por atendimento odontológico influenciaram a chance do desfecho, e indivíduos que nunca procuraram o serviço apresentaram maior chance do evento (OR 2,72; p<0,01). Pessoas idosas sem plano odontológico apresentaram chance 87% maior de edentulismo (OR 1,87; p<0,01)

Entre as características individuais, o sexo feminino (OR 1,24; p<0,01) e a idade acima da mediana (≥69 anos; OR 1,74; p<0,01) mostraram-se com maiores chances independentes. A escolaridade revelou-se o determinante com maior tamanho de efeito, com aumento progressivo da chance entre indivíduos com menor nível educacional; aqueles sem escolaridade apresentaram chance cerca de quatro vezes superior à daqueles com ensino superior (OR 4,25; p<0,01).

## DISCUSSÃO

Embora os inquéritos nacionais de saúde bucal apontem tendência de maior preservação dentária entre idosos^21^, esse avanço não tem ocorrido de forma equitativa. A persistência de diferenças por escolaridade e raça/cor sugere que eventuais ganhos populacionais coexistam com desigualdades estruturais entre subgrupos, mediadas por fatores socioeconômicos e territoriais^22,23^.

A perda dentária configura-se como fenômeno complexo, resultante da interação entre fatores sociais, ambientais, clínicos e comportamentais^24^, com repercussões que extrapolam a cavidade bucal^25^. Estudos prévios com dados do SB Brasil 2003 já demonstravam o efeito do acesso aos serviços odontológicos e do padrão assistencial sobre a perda dentária^26^. Da perspectiva da dentição funcional^27^, persistem desigualdades segundo a posição socioeconômica, mostrando que a manutenção de 20 ou mais dentes naturais permanece socialmente distribuída^28^.

De forma consistente, a maior disponibilidade de cirurgiões-dentistas por habitante apresentou efeito protetor no modelo ajustado, atestando que a preservação dentária depende não apenas de características individuais, mas também da organização da oferta de cuidado. Assim, a perda dentária no envelhecimento deve ser compreendida no contexto das condições sociais e da estrutura dos sistemas de cuidado.

A não permanência da variável raça/cor no modelo ajustado pode refletir mediação por fatores socioeconômicos e territoriais, não devendo ser interpretada como ausência de desigualdade racial. Ainda assim, a robustez amostral do SB Brasil 2023 fortalece a validade dos achados.

Na expressão do sorriso, o retrato da desigualdade social é estampado. Relatório produzido pelo Observatório Brasileiro das Desigualdades mostra que o expressivo déficit de serviços públicos é sentido pelos mais pobres, afetando as condições mais básicas de cidadania em todas as etapas da vida^29^.

Nesse contexto, analisar a perda dentária entre idosos constitui um importante indicador do funcionamento do sistema de saúde bucal de um país. As projeções construídas para o Brasil dão conta de que o edentulismo alcançará 85,96% das pessoas idosas em 2040, o que representa mais de 64 milhões de pessoas^21^.

Fagundes et al.^30^ destacam a importância de compreender os macrodeterminantes da saúde para combater disparidades e promover equidade. Os autores analisaram a distribuição do edentulismo em cinco dimensões entre 2000, 2010 e 2019 em 204 países e territórios. Entre pessoas com 55 anos ou mais, houve aumento nas taxas de prevalência, incidência e anos vividos com incapacidade relacionados ao edentulismo em países de alto Produto Interno Bruto (PIB) de 2010 a 2019, com desigualdades persistentes. Países com maior desenvolvimento econômico, melhor governança e políticas sociais apresentaram taxas mais elevadas de edentulismo, possivelmente por conta do modelo dominante de atenção individualizada à saúde bucal.

O enfrentamento ao edentulismo representa um desafio de expressiva magnitude, exigindo das políticas públicas uma abordagem abrangente, voltada tanto para perspectivas preventivas quanto para aspectos de garantia de acesso ao tratamento reabilitador. Entre as diretrizes colocadas para a Política Nacional de Saúde Bucal Brasil Sorridente no ano de 2004, esses aspectos foram incluídos por meio de procedimentos de recuperação parcial ou total das capacidades perdidas como resultado da doença e de reintegração do indivíduo ao seu ambiente social e à sua atividade profissional^5,31^. O fato de a Lei n^o^ 14.572, de 2023, instituir a Política Nacional de Saúde Bucal no âmbito do Sistema Único de Saúde (SUS) garantiu a inclusão da saúde bucal no campo de atuação do SUS, instrumentalizando as ações direcionadas à produção social da saúde bucal e, especificamente, às ações odontológicas em todos os níveis de atenção à saúde^32^. Apesar da oferta de próteses dentárias no SUS, o acesso a esse serviço é restrito. Os recursos públicos e os Laboratórios Regionais de Prótese Dentária (LRPD) limitam-se a realizar próteses totais, com capacidade inferior às necessidades da população^33^.

Embora o delineamento transversal limite inferências causais, a análise multinível permitiu explorar a articulação entre fatores individuais e contextuais. Contudo, outras limitações podem ser observadas, dentre elas potenciais multicolinearidades entre variáveis contextuais.

Não é natural perder os dentes ao envelhecer^34^. Os resultados aqui apresentados ilustram um panorama evitável, injusto e desnecessário de determinação social da saúde bucal. Garantir a superação de fragilidades na rede de atenção à saúde bucal que atravessam o caminho de manutenção dos dentes na cavidade bucal é prioridade na defesa de que nenhum sorriso precise ser escondido.

“O silêncio dos sorrisos perdidos” reflete, de forma analítica, como a perda dentária entre idosos no Brasil simboliza desigualdades sociais históricas. O sorriso torna-se um marcador da exclusão do cuidado em saúde e da omissão estatal, agravada por modelos assistenciais que geraram mutilações ao longo das gerações. Embora existam avanços na preservação dentária segundo o SB Brasil 2023, persistem disparidades ligadas à raça/cor e escolaridade. O edentulismo é resultado de escolhas políticas e estruturais evitáveis, e superá-lo demanda fortalecer o acesso integral e equitativo à saúde bucal, reconhecendo o sorriso como direito humano fundamental.

## APROVAÇÃO NO CEP:

O Projeto SB Brasil 2023 foi aprovado pelo Comitê Nacional de Ética em Pesquisa (Parecer no 4.823.054).
